# REPLY TO: “OPENING THE BLACK BOX OF REHABILITATION: GUIDE-REHAB AND THE ICF CLASSIFICATION”

**DOI:** 10.2340/jrm.v58.46867

**Published:** 2026-09-30

**Authors:** Paolo CAPODAGLIO, Maria Gabriella CERAVOLO, Carlotte KIEKENS, Calogero MALFITANO, Stefano NEGRINI

**Affiliations:** 1 Department of Biomedical, Surgical and Dental Sciences, University of Milan, Milan; 2 Istituto Auxologico Italiano IRCCS, Milan; 3 Department of Experimental and Clinical Medicine, Marche Polytechnic University, Ancona; 4 Laboratory of Evidence-Based Rehabilitation, IRCCS Galeazzi-Sant’Ambrogio Hospital, Milan; 5 Department of Biomedical Sciences for Health, University of Milan, Milan; 6 Azienda di Servizi alla Persona Istituti Milanesi Martinitt e Stelline e Pio Albergo Trivulzio, Milan, Italy

*To the Editor,*

We read with great interest the recent commentary by Boldrini and Zampolini (1) on “Mapping the commonalities of rehabilitation: using GUIDE-Rehab to open the black box of programmes across diverse health conditions” (2). While acknowledging the conceptually important contribution of Negrini et al. to the field of Rehabilitation, the authors touch on a few crucial issues that deserve attention while the iron is hot. They rightly highlight a fundamental flaw in rehabilitation research and practice: despite the International Classification of Functioning, Disability and Health (ICF) global mandate to include social participation and environmental context, clinical trials and daily practice tend to drift toward isolated, impairment-level targets (1). The in-hospital rehabilitation process acts as a highly supportive, barrier-free island, where patients may achieve high capacity under supervision but then experience a collapse in performance upon returning to their environments, where they face unmeasured obstacles (3).

To translate clinical capacity into real-world performance, trial designs and clinical workflows should systematically integrate multidimensional person–environment interactions. Environmental determinants are indeed major, active drivers of progression and functional decline in complex conditions, such as stroke, traumatic brain injury, and Parkinson’s disease. Standardized assessments should combine person–environment interactions (relying on patient-reported barriers and facilitators via validated instruments like the Craig Hospital Inventory of Environmental Factors or the Facilitators and Barriers Survey) with independent environmental features (such as Geographic Information Systems or wearable sensors), including life space assessment, mapping of green spaces, walkability, and transit proximity (4). Trials should formally compute the “capacity–performance gap” to infer environmental barriers and facilitators directly from patterns of functioning (4).

In line with the “One Health” paradigm, functioning, disability, and behavioural adaptation to environmental stimuli are shaped by a complex, lifelong interaction with the environment. Prospectively, the total cumulative lifetime physical, chemical, lifestyle, dietary, and social exposures, the so-called “exposome”, that interact with an individual’s gen-ome to modulate the rehabilitation outcome, should be incorporated in trial design (5). Also, because social context is a primary determinant of participation, trials should systematically monitor the patient’s immediate social ecosystem, including caregiver availability and caregiver burden, to isolate the true therapeutic effect (6).

A potential strength of the GUIDE-Rehab guideline is its explicit requirement to report Background Treatments, which prevents investigators from sweeping concurrent, non-experimental medical care under the non-replicable, unscientific rug of generic “usual care”. However, to truly open the “black box” of rehabilitation and fully exploit the potential of GUIDE-Rehab, the latter should advocate for a holistic, structured description of all of the key background treatments, including those yet so often neglected or unreported. Take as an example Rehabilitation Nutrition, not merely the conventional clinical nutrition support aiming to normalize biochemical parameters, but the nutrition care management that takes the rehabilitation plan into account. In neurological and geriatric populations, for instance, immobility, oropharyngeal dysphagia, and post-event hypercatabolism interact to initiate a self-reinforcing disability–malnutrition–sarcopenia cycle, where muscle loss and sarcopenic dysphagia compound primary motor deficits. Or, delivering an intensive, high-repetition exercise prescription or robotic-assisted gait training without adequate protein and energy substrate forces the body into muscle catabolism, blunting the anabolic response (anabolic resistance) and causing the intervention to under-deliver on its promise. If nutrition is neglected or not reported in clinical trials, unmeasured nutritional status acts as a major confounder. By explicitly reporting nutritional intake and the coordination of nutrient timing with therapy sessions, researchers can control for metabolic confounders, and ensure that functional recovery is accurately attributed.

The GUIDE-Rehab framework provides the taxonomic decomposition (targets, components, ingredients, and modifiers) required to structure these complex interconnections. By making targets, components, ingredients, background treatments, and rationale of interventions explicit, this granularity allows researchers to reproducibly describe the determinants of rehabilitation outcomes while identifying implicit or neglected elements. Although GUIDE-Rehab does not inherently close the Impairment–Functioning gap, it provides the means to unveil it. We now have a powerful, albeit demanding, tool to help bridge this gap in research and practice – the question remains whether the field is ready to rise to the challenge.
